# The Molecular-Social-Genetic Determinants of Cardiovascular Health in Pacific Islanders

**DOI:** 10.1016/j.jacasi.2024.04.012

**Published:** 2024-06-18

**Authors:** Yoon Seo Lee, George Lord, Austin Szatrowski, Zane A. Maggio, Andrew Kekūpaʻa Knutson, Jerris R. Hedges, Bohdan B. Khomtchouk

**Affiliations:** a Harvard John A. Paulson School of Engineering and Applied Sciences, Harvard University, Boston, Massachusetts, USA; b The College of the University of Chicago, Chicago, Illinois, USA; c Department of BioHealth Informatics, Luddy School of Informatics, Computing, and Engineering, Indiana University, Indianapolis, Indiana, USA; d Center for Cardiovascular Research, John A. Burns School of Medicine, University of Hawaiʻi at Mānoa, Honolulu, Hawaii, USA; e Departments of Medicine and Surgery, John A. Burns School of Medicine, University of Hawaiʻi at Mānoa, Honolulu, Hawaii, USA; f Krannert Cardiovascular Research Center, Indiana University School of Medicine, Indianapolis, Indiana, USA; g Center for Diabetes & Metabolic Disorders, Indiana University School of Medicine, Indianapolis, Indiana, USA; h Center for Computational Biology & Bioinformatics, Indiana University School of Medicine, Indianapolis, Indiana, USA; i Department of Medical and Molecular Genetics, Indiana University School of Medicine, Indianapolis, Indiana, USA

**Keywords:** cardioinformatics, health disparities, Native Hawaiians, Pacific Islanders, precision medicine

The term “Pacific Islander” typically refers to people with roots in the indigenous communities of Polynesia, Micronesia, and Melanesia, and “Native Hawaiian” specifically refers to individuals who descend from indigenous peoples who lived in the Hawaiian Archipelago before Western contact.^1^^,^^2^ As a result of their smaller population size relative to other racial and ethnic groups within the United States, and frequent aggregation with other demographics, Native Hawaiian and Other Pacific Islander (NHOPI) individuals have historically been left underrepresented in biomedical research datasets.^2^ Significant health disparities exist among NHOPI individuals relative to other populations, including higher rates of obesity, type 2 diabetes (T2D), and cardiovascular disease (CVD), in addition to their associated risk factors.^1^^,^^3^ NHOPI individuals also represent one of the fastest-growing populations in the United States and this rapid growth could intensify existing disparities unless the underlying causes are identified and effectively addressed.^1^ Consequently, there is a critical need for enhanced research, more innovative drug discovery, and targeted clinical interventions tailored to these underrepresented populations.

## The NHOPI Data Disparity and Consequences of Demographic Aggregation

Historically, NHOPI individuals have received relatively less attention in cardiovascular health research.^1^ In addition, within the NHOPI racial category, ethnic groups such as Hawaiian, Samoan, Tahitian, and Māori individuals are seldom delineated and thereby understudied. To explore this unmet need further, we systematically reviewed existing human health data sets and large-scale epidemiological cohort studies across different biological databases and biobanks covering various cardiovascular disease phenotypes to assess current NHOPI representation therein (Table 1). All major CVD-relevant studies were included, even if zero NHOPI participants were recorded, and other studies were included if they attempted to disaggregate the NHOPI population from the larger "Asian" umbrella term. Studies with zero-recorded NHOPI participants may have either aggregated data with other recorded categories (eg, “Asian”) or have no NHOPI-identifying participants. With such a paucity of biomedical data, reliable analyses of cardiometabolic risk are exceedingly difficult and represent an active area of computational research.

**Table 1:** Summary of NHOPI Data in CVD-Relevant Studies

Data Set/Study Name	Total Count NHOPI (n)	Total Participant Count
NIH *All of Us* Research Program	1,112	545,600
Kaiser Permanente Research Bank	4,252	325,124
UK Biobank	754	500,000
PAGE: Multiethnic Cohort	2,502	215,000
University of Hawaii Multiethnic Cohort	13,971	215,251
Million Veteran Program (MVP)	3,615	650,000
Framingham Cohort (FC)	<50	5,209
Multi-Ethnic Study of Atherosclerosis (MESA)	0	6,814
Atherosclerosis Risk in Communities (ARIC) Cohort	<48^a^	15,792
Cardiovascular Health Study (CHS)	0	5,888
Women’s Health Initiative (WHI)	837	>93,000
Indiana University Biobank	51	44,654
Molokai Heart Study^b^	257	257
Cardiovascular Risk Clinic Program^b^	855	862
Kā-HOLO Project^b^	250	250
Cardiovascular Disease among Asians and Pacific Islanders (CASPER)^b^	14,295	840,718
Malama Pu‘uwai Study^b^	127	127
Pacific Islands Cohort on Cardiometabolic Health Study (PICCAH)^b^	Aiming for 1,200	Aiming for 1,200
Total (excluding PICCAH)	42,878 (excluding FC, ARIC)	3,464,546

Data in all studies are anonymized, so potential overlap could not be assessed. Contains the total NHOPI count and total participant count of several major CVD-relevant studies.

CVD = cardiovascular disease; NHOPI = Native Hawaiian and Other Pacific Islander.

a Including American Indian, Alaskan Indian, and/or Asian.

b Designed to focus on NHOPI individuals.

Of the 18 data sets recorded in Table 1, 6 were targeted Hawaiʻi-based health projects that prioritized recruiting NHOPI participants. Excluding these 6 NHOPI-focused studies, there were a total of 27,094 NHOPI individuals within the 12 remaining data sets, constituting approximately 1% of the total participants within these other biomedical initiatives. However, including these 6 NHOPI-focused cohorts, there were a total of 42,878 participants properly recorded as NHOPI, constituting 1.24% of the 3,464,546 total participants who were assessed. In addition, NHOPI individuals face a unique challenge to health equity, as their smaller populations make it more difficult to recruit participants for clinical cohort studies. To address this, NHOPI-focused initiatives like the Wahine Heart Wellness Program and the Pacific Island Health Care Project are crucial, as they not only help increase overall representation and inclusion but also play a key role in identifying factors contributing to health disparities among NHOPI individuals.

## Using Genomic Data for NHOPI Disaggregation in Population Genetics Research

NHOPI individuals are underrepresented and seldom disaggregated in major biobanks and epidemiological cohort studies, which can mask genetic heterogeneity of potential clinical relevance. To better understand this, we used dimensionality reduction methods such as principal component analysis and machine learning techniques like Neural ADMIXTURE for genetic clustering. We selected a sample of 2,298 individuals from the National Institutes of Health (NIH) *All of Us* Research Program (AoU) database, where the self-identified races included Asian, Black or African American, Middle Eastern or North African, White, NHOPI, and “More than one population.” Before our analysis, we performed a standard genomic quality control for variant missingness, Hardy-Weinberg equilibrium, and linkage disequilibrium.

**Figure 1:**
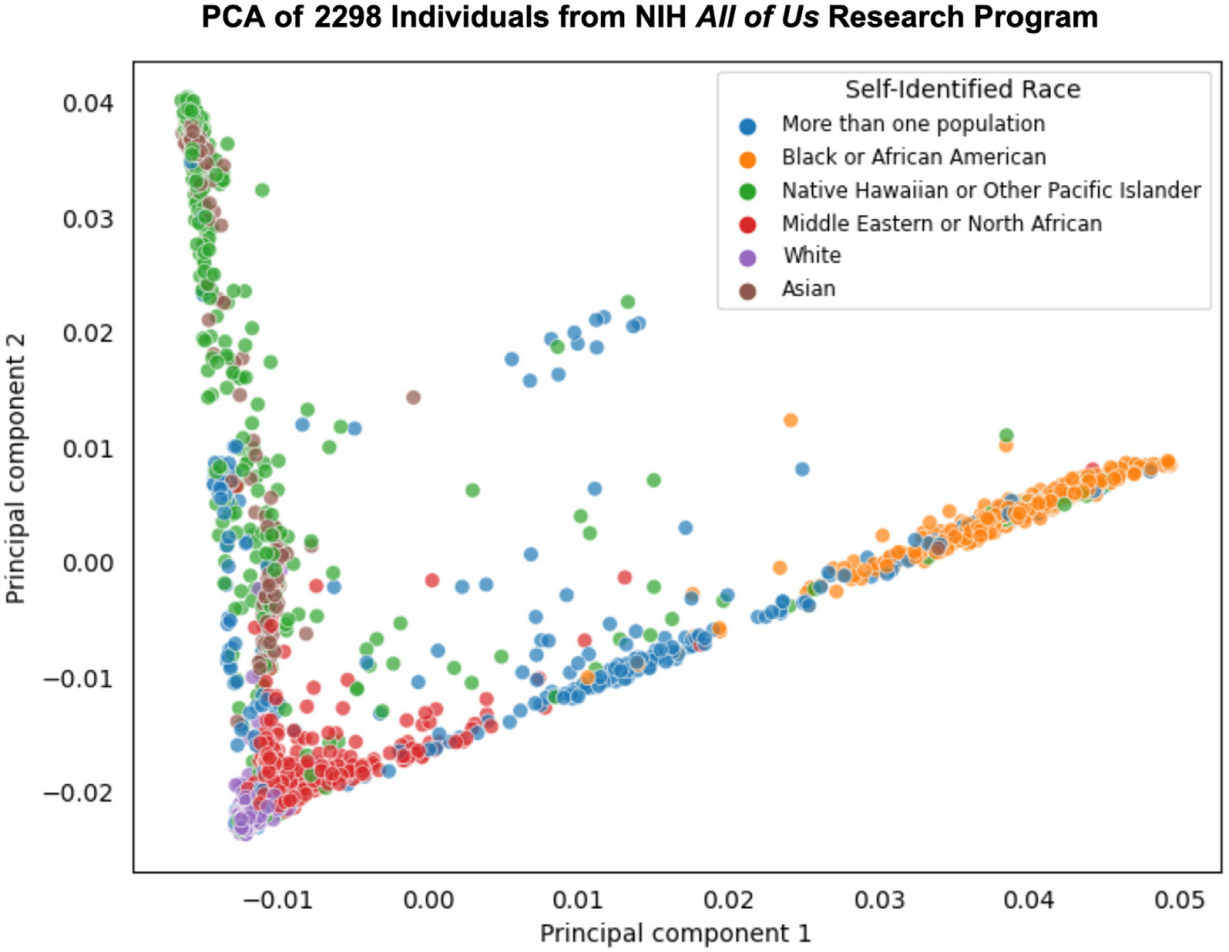
PCA of NIH *All of Us* Research Program Sample Cohort Principal component analysis (PCA) of 857,883 autosomal single nucleotide polymorphisms for 2,298 individuals from the *All of Us* biobank. After cohort selection, standard genomic quality control, followed by PCA, was performed with PLINK 1.9. Individual clusters, colored by the self-identification of each individual, indicate population heterogeneity in marker genotypes between clusters. The delineated Native Hawaiian and Other Pacific Islander (NHOPI) and Asian clusters indicate the need to disaggregate Asian and NHOPI individuals in association analysis regressions for cardiovascular diseases.

Principal component analysis of 857,883 autosomal variants from this sample into 2 principal components revealed distinct population clusters (Figure 1). When projected onto the first 2 principal components, clusters of similarly colored points indicate genetic similarity within groups, and separations indicate genetic differences. Noticeably, the NHOPI individuals form a cluster as well, indicating a distinct population from other ancestral groups, including Asian individuals.

**Figure 2:**
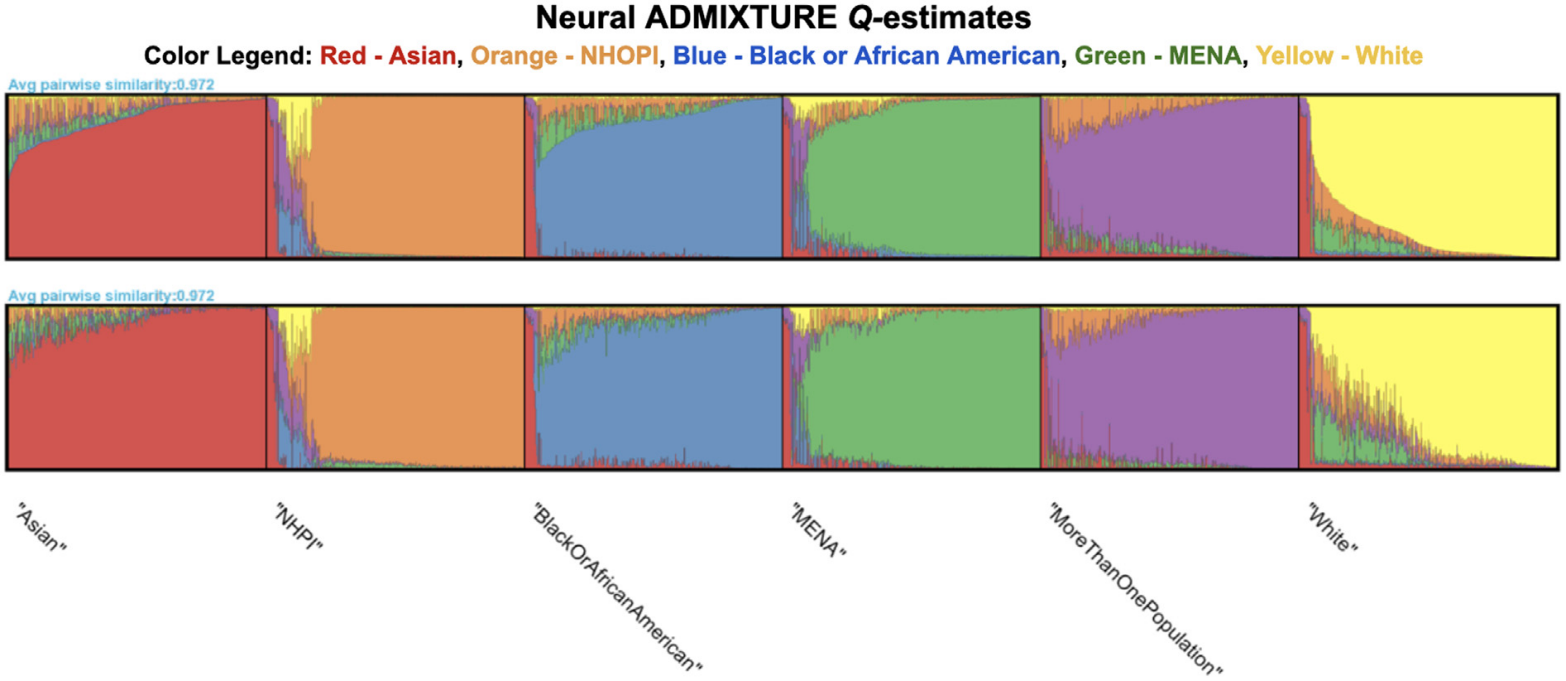
Neural ADMIXTURE *Q-*estimates for *All of Us* Sample Cohort Cluster assignment across 2 modes from 5 *Q-*estimates for 2,298 individuals in the National Institutes of Health *All of Us* Research Program computed by Neural ADMIXTURE. Standard genomic quality control was conducted in PLINK 1.9, and *Q-*estimates were computed by the Neural ADMIXTURE software package. Each vertical bar corresponds to an individual, and the proportion of each color corresponds to their proportion of ancestry from that cluster. The strong assignment of each ancestry group in the data set to the colored clusters indicates population-scale heterogeneity between Native Hawaiian and Other Pacific Islander (NHOPI) and Asian individuals, and the importance of data disaggregation in genetic association analysis regressions for cardiovascular disease. MENA = Middle Eastern or North African.

Neural ADMIXTURE fractional ancestry assignments (*Q*-estimates) were generated for each individual in the sample (Figure 2). Multimodality in *Q*-estimates was visualized and identified using the *pong* software package, in which the estimates may differ nontrivially even with the same input genotype data. Two major modes were encountered across 5 runs, each with a 97.2% degree of pairwise correspondence, indicating little multimodality across *Q*-estimates in each of the 2 major modes. In addition, in both major modes, each ancestry group showed a high degree of membership to its corresponding cluster, indicating that the *Q*-estimates effectively assign each person (depending on ancestry group) to their corresponding cluster.

Taken together, dimensionality reduction techniques (eg, principal component analysis) and machine learning methods for genetic clustering (eg, Neural ADMIXTURE) show significant heterogeneity across ancestry groups in the AoU sample, thereby further indicating that disaggregation between NHOPI, Asian, and other ancestral groups is essential in future cohort studies and biobanks, especially for genetic studies of CVD in these populations.

## Elevated Risk Factor Levels Among NHOPI Populations

In addition to this underrepresentation, NHOPI populations carry elevated risk for cardiometabolic disease. Based on analyses conducted in the NIH AoU Research Program, we found that NHOPI patients residing in the United States have significantly lower high-density lipoprotein-cholesterol (HDL-C) levels and higher triglyceride levels than other racial groups, putting them at elevated risk for developing hypertension, T2D, and heart disease. In particular, our analyses revealed that although HDL-C in White individuals was 55.69 mg/dL (95% CI: 55.55-55.83 mg/dL), HDL-C in NHOPI individuals was significantly lower (*P* < 0.05) at 52 mg/dL (95% CI: 49.33-54.59 mg/dL). HDL-C was higher, approaching the threshold for significance, in all other groups represented in the AoU cohort. Similarly, triglyceride levels in NHOPI participants within AoU were elevated relative to all other populations except Hispanic individuals (128 mg/dL, 95% CI: 118-137 compared with, eg, 120 mg/dL, 95% CI: 119-120 in White individuals [Table 2]). No significant differences were observed in low-density lipoprotein-cholesterol in this comparative analysis of AoU data. Furthermore, we compared systolic blood pressure across demographics using the AoU data sets and found a similar health risk disparity: 130 mm Hg (95% CI: 128-133 mm Hg) in the NHOPI sample, which was significantly higher (*P* < 0.05) than all other groups except in Black individuals (Table 2).

**Table 2:** Comparison of Clinical Risk Factors Across Demographic Groups Using NIH *All of Us* Research Program Data

Group	HDL-C (mg/dL)		LDL-C (mg/dL)		Triglycerides (mg/dL)		Blood Pressure (mm Hg)	
	Mean	95% CI	Mean	95% CI	Mean	95% CI	Mean	95% CI
NHOPI	51.96	49.33-54.59	98.18	93.22-103.14	127.79	118.11-137.47	130.1	127.63-132.57
Asian	56.49	55.9-57.08	104.48	103.33-105.63	120.71	118.49-122.93	121.49	120.93-122.05
Black	53.72	53.48-53.96	101.71	101.21-102.21	109.88	109.04-110.72	131.69	131.4-131.98
Hispanic	50.23	50-50.46	101.4	100.86-101.94	135.25	134.19-136.31	125.7	125.42-125.98
MENA	54.19	53.17-55.21	103.87	101.8-105.94	116.86	113.1-120.62	121.79	120.78-122.8
White	55.69	55.55-55.83	103.2	102.94-103.46	119.55	119.05-120.05	126.58	126.44,126.72

HDL-C = high-density lipoprotein-cholesterol; LDL-C = low-density lipoprotein-cholesterol; MENA = Middle Eastern or North African; NHOPI = Native Hawaiian and Other Pacific Islander; NIH = National Institutes of Health.

## Health Disparities in NHOPI Cardiometabolic Disease Burden

As a result of multiple risk factors, NHOPI individuals are disproportionately burdened by cardiometabolic diseases.^1^^,^^3^ Recent studies have shown that NHOPI patients have high rates of undiagnosed T2D as well as high rates of diagnosed metabolic disease: 19% to 22% for T2D, and 16% to 35% for impaired glucose tolerance.^1^^,^^2^ One recent prospective study found a prevalence of T2D as high as 57.9%, noting that many cases were previously undiagnosed.^1^ In addition, a study in Oʻahu found that among 562 patients hospitalized with acute intracerebral hemorrhage (stroke), NHOPI patients were younger than Asian and White patients (*P* < 0.0001) while also having a higher prevalence of diabetes and hypertension.^3^ NHOPI individuals have been found to face higher prevalence of CVD risk factors and die from CVD-related complications at a younger age when compared with White patients.^3^

## Unique Genetic Factors Affecting Efficacy of Pharmaceutical Treatments

In addition to being underrepresented and experiencing unique incidences of cardiometabolic diseases, NHOPI individuals have also shown distinct responses to standard pharmaceutical treatments. For example, a 2013 study on the dosing of warfarin in Hawaiʻi determined that ethnicity should be taken into account because ethnically stratified genetic variants in the *CYP2C9* and *VKORC1* genes affected the drug’s metabolism.^4^ The study included patients of Asian, Native Hawaiian, Portuguese, and non-Portuguese White descent who were already on warfarin, a common anticoagulant. The results showed that NHOPI patients, given their *CYP2C9* and *VKORC1* genotypes, required significantly lower dosages compared with White patients.^4^

*CYP2C19*, a P450 protein involved in drug metabolism, also has ethnically stratified effects. Its ∗2 allele, a loss-of-function variant, is associated with increased risk for adverse cardiac events in patients treated with clopidogrel, an antiplatelet medication. This allele was found to be far more common in Pacific Islander individuals residing in New Zealand (45% heterozygosity) than in other New Zealand–based populations, including Māori (24%), Asian (29%), and European (15%) individuals.^5^ Subsequent pharmacoeconomic analysis found that a genetically guided clopidogrel-vs-prasugrel treatment decision was cost-effective, especially for Māori and Pacific Islander patients, as it reduced incidence of adverse events and brought down hospitalization costs.^5^ Before these findings came to light, Bristol Myers Squibb marketed clopidogrel (brand name: Plavix) to Hawaiʻi-based physicians for decades and, in 2020, a Hawaiʻi court ordered Bristol Myers Squibb to pay $834,012,000 in penalties for violating the state’s unfair and deceptive practices laws.^6^

In addition to warfarin and clopidogrel, classic cardiovascular disease management tools like angiotensin-converting enzyme inhibitors and beta-blockers may show increased efficacy if used differently in non-Caucasian ethnic groups, particularly NHOPI individuals, as dosage sensitivity may vary (Table 3). Table 3 lists an array of pharmaceutical drugs prescribed for CVD treatment that have been found to potentially elicit differential responses in NHOPI patients.

**Table 3:** Drug Response Differences in NHOPI Individuals: A Summary of the Key Findings of Studies That Have Investigated Drug Response Differences in NHOPI Individuals Compared With Other Groups

Drug or Drug Class	Potential Differences in Response	References Cited
Statins	Genetic variations alter response so some populations (eg, Samoan individuals) may respond more favorably to lower doses	AlAzzeh and Roman,^7^ Birmingham et al^8^
Warfarin (trade names: Coumadin, Jantoven)	NHOPI individuals may require different doses of warfarin because of genetic variations affecting drug metabolism	Tatsuno and Tatsuno^4^
Clopidogrel (trade name: Plavix)	Provides little-to-no benefit in NHOPI populations	Hawaii Attorney General News Release^6^
ACE inhibitors	ACE inhibitors may have higher efficacy in Asian populations compared with others, as a first-line or second-line agent	Gupta et al^9^
Beta-blockers	Asian populations may require lower doses of beta-blockers because of increased sensitivity to the drugs	Gupta et al,^9^ Altshuler et al^10^

ACE = angiotensin-converting enzyme; NHOPI = Native Hawaiian and Other Pacific Islander.

To determine whether there were disparities not just in drug response, but also in drug prescriptions, we performed a comparative analysis of prescription rates in NHOPI individuals relative to non-NHOPI individuals for the treatments listed in Table 3 using the NIH AoU Research Program cohort. For each drug analyzed, where prescription information was readily available in the database, the NHOPI population had a lower percentage of individuals prescribed each drug compared with non-NHOPI individuals (Table 4), despite the observation that the corresponding risk factor (eg, dyslipidemia or hypertension) was often higher (Table 2).

**Table 4:** NIH *All of Us* Research Program Prescription Rate Differences in NHOPI Individuals

Drug Name (Class)	No. of NHOPI Prescribed	NHOPI Percentage Prescribed	No. of Non-NHOPI Prescribed	Non-NHOPI Percentage Prescribed
Rosuvastatin (Statin)	37	3.32	23,239	5.80
Warfarin	65	5.84	30,588	7.63
Perindopril (ACE inhibitor)	0	0	36	0.01
Atenolol (BB)	34	3.05	18,155	4.53
Metoprolol (BB)	230	20.7	86,797	21.67
Propranolol (BB)	26	2.34	14,420	3.60

n = 1,112 NHOPI, n = 400,556 non-NHOPI.

ACE = angiotensin-converting enzyme; BB = beta-blocker; NHOPI = Native Hawaiian and Other Pacific Islander; NIH = National Institutes of Health.

## The NHOPI Diaspora and Emerging NHOPI Communities Across the Continental United States

The sprawling demographic landscape of NHOPI individuals in the United States is actively undergoing significant changes, with immediate clinical implications for cardiovascular and metabolic care. To analyze this dynamic, we calculated the estimated percentage of NHOPI individuals by U.S. county and the total movement of NHOPI individuals into each county between 2016 and 2021 from the ACS-5 U.S. Census data. The data showed that NHOPI individuals constitute a minority within each county in Hawaiʻi, particularly in Honolulu County, where only 26.65% self-identify as NHOPI. Moreover, only 36% of NHOPI individuals moving to counties in Hawaiʻi came from other U.S. states, with the remainder coming from other counties within Hawaiʻi.

In addition, the data reveal growing NHOPI communities in the western, midwestern, and southern United States, as well as within the State of Alaska. In the continental United States, Clark County (Nevada) and Pierce County (Washington) were the 2 counties that saw the most NHOPI individuals moving in from out-of-state, potentially because of factors such as more diverse employment opportunities in major cities like Las Vegas. Furthermore, when excluding Hawaiʻi, the top 10 U.S. destinations for NHOPI individuals account for approximately 25% of NHOPI movement from 2016 to 2021. In fact, the top 5 destination counties experienced a greater combined influx of NHOPI individuals, totaling 5,854 individuals, compared with all of Hawaiʻi, which saw 4,716 individuals. This trend is likely exacerbated by the widening wealth gap in Hawaiʻi, prompting natives and longtime locals to move to areas with a more affordable cost of living. The significant expansion of NHOPI communities in counties across the United States, beyond Hawaiʻi, highlights the urgent need to extend precision medicine initiatives to NHOPI individuals in these regions.

## Conclusions

The historical underrepresentation of NHOPI individuals in biomedical data set inclusion has perpetuated significant cardiometabolic health disparities over time. NHOPI individuals are currently one of the fastest-growing populations in the United States, which instills urgency for increased health equity and diversity in research and clinical trials to address issues that often arise from the erroneous aggregation of NHOPI data with other demographic groups. Addressing these clinical unmet needs in NHOPI population health necessitates significant community intervention and efforts to remedy the underlying disparities including, but not limited to increased federal funding for NHOPI-focused research, changes in public policy, more accurate demographic categorization, genomic clustering, and other precision medicine approaches, as well as the implementation of pharmacogenomic and culturally sensitive treatment strategies in order to improve NHOPI health care outcomes on a local and global scale.

## Funding Support and Author Disclosures

Research reported in this publication was supported by the National Institute of Diabetes and Digestive and Kidney Diseases (NIDDK) of the National Institutes of Health (Bethesda, Maryland) (R01DK132090 to Dr Khomtchouk). The authors have reported that they have no relationships relevant to the contents of this paper to disclose.
